# Protein Complex Formation: Computational Clarification of the Sequential versus Probabilistic Recruitment Puzzle

**DOI:** 10.1371/journal.pone.0055046

**Published:** 2013-02-01

**Authors:** Manuel Schölling, Stefan Thurner, Rudolf Hanel

**Affiliations:** 1 Section for Science of Complex Systems/CeMSIIS, Medical University of Vienna, Vienna, Austria; 2 Santa Fe Institute, Santa Fe, New Mexico, United States of America; Semmelweis University, Hungary

## Abstract

Our current view on how protein complexes assemble and disassemble at promoter sites is based on experimental data. For instance this data is provided by biochemical methods (e.g. ChIP-on-chip assays) or GFP-based assays. These two approaches suggest very different characteristics for protein recruitment processes that regulate and initiate gene transcription. Biochemical methods suggest a strictly ordered and consecutive protein recruitment while GFP-based assays draw a picture much closer to chaotic or stochastic recruitment. To understand the reason for these conflicting results, we design a generalized recruitment model (GRM) that allows to simulate all possible scenarios between strictly sequential recruitment and completely probabilistic recruitment. With this model we show that probabilistic, transient binding events that are visible in GFP experiments can not be detected by ChIP experiments. We demonstrate that sequential recruitment processes and probabilistic recruitment processes that contain “shortcuts” exhibit periodic dynamics and are hard to distinguish with standard ChIP measurements. Therefore we propose a simple experimental method that can be used to discriminate sequential from probabilistic recruitment processes. We discuss the limitations of this method.

## Introduction

Understanding the regulatory processes which govern cells in a systemic way is an increasingly important challenge in biology and medicine [1]–[5]. One particular challenge of importance within this rapidly evolving field of research is to understand how *transcription* is modulated [6]–[9] and thus how abundance of mRNA is controlled on the side of mRNA production (as compared to mRNA degradation [10], [11]). A body of research (e.g. [12]–[16]) has contributed to enhance the understanding of transcription initiation and control, extending the basic principle of promoter elements serving as “anchors” for the assembly of intermediate protein complexes and the general transcriptional machinery. These add-ons to the basic picture, which traditionally consists of a strictly sequential recruitment of elements from the promoter over the formation of the pre-initiation complex (PIC) to initiation of transcription by RNA polymerase II (PolII), include cis- and trans-regulatory elements, co-regulators and chromatin remodelers [17].

For understanding the dynamics of the transcription initiation and modulation, mainly nuclear receptors have been used as models [17]. Biochemical experiments (e.g. ChIP assays for the 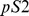 gene [12]) have been used to analyze the transcription initiation process. Despite all progress in elucidating regulatory details, the predominant model for interpreting experimental data relies on the concept of strictly ordered *sequential recruitment* processes (SR) [12], [16], [18], [19].

SR became challenged with the *green fluorescent protein* (GFP) revolution. Particularly GFP-based assays like *fluorescence recovery after photobleaching* (FRAP) [20]–[22] draw a far more dynamic picture of protein recruitment, the probabilistic recruitment (PR) scenario. PR suggests stochastic models for assembling the transcriptional machinery (e.g. [23], [24]).

While it is in principle possible that different promoter sites show different *recruitment characteristics*, it is obviously true that the two scenarios, SR and PR, of protein recruitment can not be true at the same time for the same recruitment process. Several attempts have been made to reconcile both scenarios (e.g. [17], [24], [25]). One strategy is to find reasons why biochemical methods, like ChIP-based assays, may overestimate the regularity of the recruitment process and GFP-based assays may overestimate the stochasticity [25]. Another strategy [17], [24] is basically to reinstate the SR scenario by assuming a *transcriptional ratchet* at work that progresses only when the right binding partners or *allosteric interactions* initiate an irreversible functional change of the assembled protein complex giving the process a definite direction. Other random interactions (as seen by GFP-based assays) simply are rationalized away as non-functional reversible interactions. Basically the only stochastic element that remains in this approach consists of stochastic waiting times between functional state changes [18].

Nonetheless, it is known that the recruitment process can partially take place in a non-sequential, probabilistic way. For example Rafalska-Metcalf et al. (2010) demonstrated that certain transcription factors can replace the sequential binding of a series of other proteins for VP16- and p53-induced transcription in transgenic human osteosarcoma cells. Thus these transcription factors can act as “shortcuts” in the recruitment process. Another example was shown by Esnault et al. (2008): According to their results the PIC can be formed by several pathways that differ in the sequence of binding of general transcription factors.

Different models have been proposed to simulate recruitment processes (e.g. [18], [26]). Lemaire et al. (2006) formulated a model to simulate recruitment in a strictly sequential order with the aim to reconstruct the binding pattern of proteins and protein complexes. This model proposed a cyclic protein binding pattern for ChIP data. D’Orsogna and Chou (2005) modeled complex formation by ligand binding and compared the mean first-passage time of the sequential and *combinatorial* recruitment scenarios.

Since current attempts to reconcile the SR and the PR scenario remain incomplete, we re-analyze the problem by designing a generalized recruitment model (GRM) that allows to simulate all possible scenarios ranging between strictly sequential and completely probabilistic. We use the model to predict the result of ChIP experiments based on different underlying recruitment models and compare the predicted characteristics with experimental data. We show that recruitment processes that deviate strongly from the SR scenario contradict the ChIP experimental facts. We also show three additional things: (i) If each step in a cyclic recruitment process is reversible (where the forward transition rate is larger than the backward transition rate) then the ChIP signal of the resulting PR process is equivalent to a SR process with modified period. (ii) If a PR process can be obtained from a SR process by adding a few additional *long-range* transitions (i.e. opportunities for parts of the assembling protein complex to form off-site or to break-off), which allow the recruitment process to progress or regress substantial parts of the transcription cycle in one step, then ChIP will exhibit oscillatory dynamics that can easily be confounded with pure SR. (iii) We show how ChIP can in principle be used to test whether a recruitment process has such long-range transitions. A negative result however does not exclude the existence of such transitions, but implies that the deviations from the SR process are negligible and therefore the process can effectively be treated as a SR process.

Once it is known that a specific promoter-site recruits proteins strictly sequentially, then methods as e.g. described in [18] can be used for inferring the protein recruitment process. If one knows on the other hand that long-range transitions exist then those have to be incorporated in model-based inference schemes (which goes beyond the scope of this paper) and methods designed for SR will predict wrong recruitment patterns.

## A Model for Sequential and Probabilistic Recruitment

**Figure 1:**
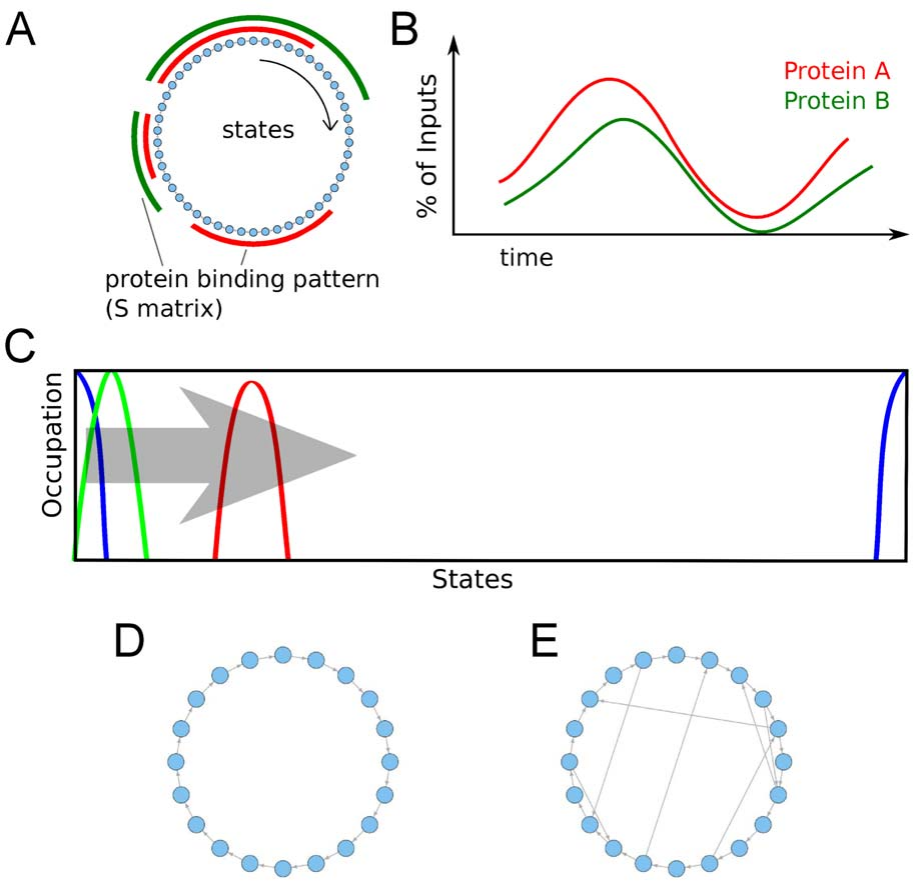
Schematic visualization of the model. (a) The recruitment process is represented by a number of states (blue nodes) that are connected by transitions (here shown for SR). The 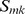 matrix determines whether a protein is present (red and green bars) in a state or not. (b) Using the GRM the resulting ChIP signals for the proteins are predicted. (c) Schematic occupation of states for Gaussian initial conditon (blue) and two later time points (green and red). (d) Recruitment network for the SR process (0 shortcuts). All states are only linked to their unique successor states in a circular manner. (e) Recruitment network for a PR process with 7 shortcuts.

We model recruitment in a more general way than [18], [26] by generalizing the approach presented in [18]. We assume that the recruitment process can be characterized by “states” 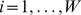, which form the 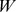 nodes of a recruitment network (Fig. 1a). Links between these nodes characterize possible transitions between these recruitment states. Intuitively such transitions between states can be understood as the addition or removal of a protein to the recruiting protein complex, as methylation or deacetylation events or even as conformal changes of the three dimensional molecular structure. Yet, only a subset of 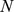 such events can be detected in ChIP experiments and can be regarded as “landmarks” in the recruitment process, then 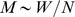 intermediate states on average lie inbetween these landmarks. As a consequence, if state-transtions follow an exponential distribution, transitions between landmark states follow a Poissonian distribution (compare Eq. 8).

ChIP experiments average over samples composed of many cells. Each cell on its own may be in a certain state 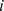. The population of cells in a sample, will in general be distributed over all states. If however, recruitment to the promoter has to be initiated by some signal (e.g. by estradiol for the 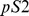 gene [12]), it can be expected that directly after initiation, the initial state distribution of a synchronized cell population will form a sharp peak around the cleared promoter state 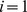. The initial distribution of cells gets denoted by 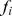. We want to know how the initial distribution 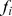 evolves over time once transcription has been initiated (Fig. 1c). We design a *generalized recruitment model* (GRM) in the following way: The waiting time between possible transitions 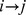 from state 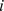 to 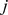 is considered as a probabilistic event drawn from an exponential distribution
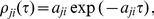
(1)where 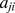 characterizes the transition rate from 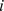 to 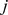. If 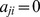 then a transition from 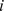 to 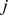 is impossible. The probability not to find a transition 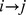 within the time-interval 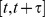 is given by



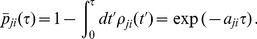
(2)The effective probability for a transition 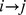 is therefore
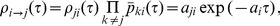
(3)where 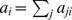. Given that 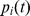 is the probability to find a cell in state 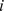 at time 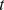 and by considering the number of transitions that arrive and leave state 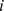 within a small time span 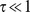, the differential equation
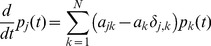
(4)holds, where 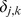 is the Kronecker symbol, which is 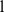 if 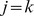 and 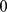 otherwise. Depending only on the transition rates 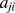, Eq. (4) governs all possible recruitment processes.

The information whether a protein 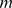 is present in a state 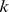 is expressed by a matrix 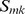: If the value of 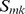 is 1 then the protein 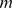 is bound in the state 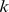 of the recruitment process, if 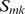 is 0 then the protein 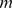 is not bound in state 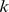. Note that the matrix 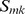 is constant over time. Since a huge number (typically 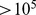) of cells is used in ChIP experiments [12], 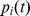 can be used to estimate how the cells’ states are distributed in a sample. Therefore, we can predict the “concentration” 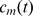 (i.e. percentage of inputs) at time 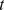 for protein 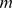 that would be measured in a ChIP experiment (compare Fig. 1a,b). Using the 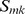 matrix we get
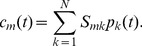
(5)


SR is represented in this model by states that are circularly linked to their successor states (Fig. 1d). Thus, for SR the 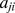 form a sub-diagonal matrix. To implement PR processes, transitions are added to this model, so that 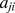 has also off-diagonal entries. These additional transitions can point either forward or backward in the recruitment process (Fig. 1e). Depending on the number 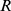 of states such transitions 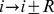 can skip without notably adding to diffusive broadening of observed singals 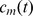 over observation time 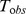 we distinguish between *short-range* and *long-range* transitions as described below in more detail.

We now analyze basic properties of the GRM (Eq. 4). First we show that PR scenarios, where the forward transitions are made reversible by backward transitions, are practically indistinguishable from SR processes by ChIP experiments. We then discuss PR processes obtained from SR processes by adding shortcuts, i.e. long-range transitions. The resulting ChIP signal of a PR process can also exhibit oscillatory dynamics. From observing periodic signals 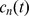 alone it is hardly possible to distinguish whether the source of the signal is SR or PR. Finally, we describe a method that allows to test whether a SR process or a PR process with shortcuts is present and comment on the limitations that may arise in a practical application of this test.

### Sequential Recruitment

For SR a state 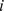 is only linked to its successor state 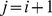 (Fig. 1d). Thus, 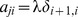 and one recovers the model used in [18], with a transition rate 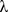.

**Figure 2:**
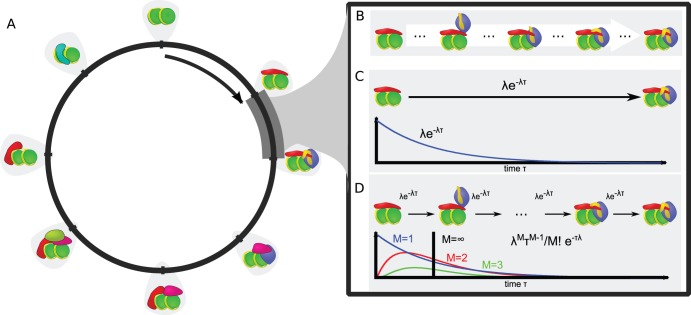
Schematic visualization landmarks and intermediate steps. (a) Using ChIP only a subset of events (landmarks) can be detected. (b) Binding events of proteins, for instance, are accompanied by conformational changes. ChIP is not sensible to these binding events and thus these intermediate processes are not reflected in the ChIP signal. (c,d) Transitions between landmarks modeled without (c) and with (d) intermediate steps: If several intermediate steps are included in the model, the transitions between landmarks become Poissonian instead of exponentially distributed. For a large number of intermediate states – as suggested by experimental ChIP data (see text) – this converges against a delta distribution.

This concept of a state also includes all substeps interpolating between the 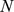 landmark events. For simplicity we assume that each of the 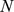 landmark events gets interpolated by 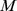 substeps and the whole number of states of the model is 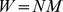 (Fig. 2). To see the effect of a large number of interpolating steps we look at the limit 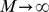. With a set of new variables, 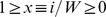 (the state 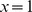 has the successor state 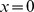), it follows that 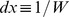, 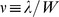, 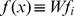, and 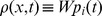. Given these substitutions Eq. (4) can be written in continuous form as
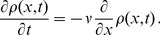
(6)


This equation has solutions of the form
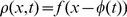
(7)with 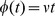. This means that in the limit 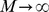 the initial distribution 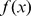 propagates periodically over the sequential states with frequency 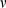. For a SR process the time series produced with ChIP will therefore show periodic dynamics of 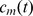. The resulting waiting times between landmark events are not exponentially distributed anymore but are given by
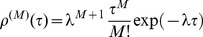
(8)and the expectation value of transition time between the 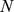 landmarks
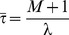
(9)defines 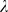. In the limit 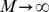 this expression converges to a delta distribution and recruitment starts to work like a clockwork rather than a stochastic process. If the limit 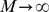 is not fulfilled, then the initial distribution smears out with time. However, ChIP data for the 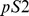 gene [12] shows that recruitment processes exist where this spread can be neglected within the typical experimental observation time in which case 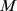 needs to be large.

### Probabilistic Recruitment

Now consider a SR process where the transitions are reversible. For simplicity we assume that the transition rates are constant. We denote the forward transition rates by 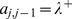 and the backward transition rates by 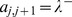 and assume that 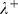 is larger than 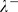. Thus, Eq. (4) states
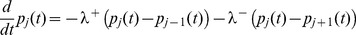
(10)and for large 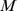 the process can be approximated by a Fokker-Plank equation
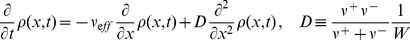
(11)with the flow term 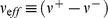, 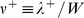, 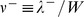 and the diffusion constant 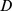. Due to diffusion the initial condition of the process will get broader by 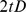 over time 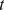. In particular, after the experimental observation time 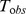 the width of the initial distribution 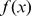 increases by 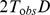. If diffusion observed for experimental data is negligible (which is the case for instance for 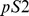 data) then 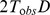, i.e. 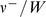, needs to be small. This means that if 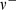 is of the same order as 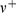 (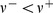), then reversible transitions only affect the effective frequency 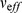 of the process while diffusion remains negligible. Moreover, if 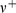 and/or 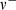 depend on the position 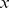 in the recruitment process then different parts 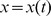 of the recruitment process are traversed with different local frequency 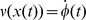.

Next, we consider transitions 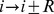 (linking forward and/or backward). Suppose such transitions are added to the SR scenario for a large fraction of states 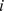 all over the recruitment cycle – analogous to adding reversible transitions as discussed above. If those transitions have rates comparable in magnitude to 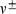 or smaller, their effect can be split into a part that modifies the effective (local) transition rate 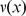 and a part 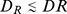 that contributes to the effective diffusion constant. Again, if no noticeable diffusive spreading of the initial condition is observed then 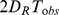 has to remain sufficiently small. We call the range of 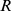 where this is true *short-range*. Conversely, we call transitions that do not fulfill this criterium *long-range.* A process where the ChIP signal shows no notable diffusion therefore can at best only contain a small number of these long-range transitions.

Recruitment processes containing long-range transitions (shortcuts) represent the last scenario we are interested in. Note that for our computer simulations we assume (for reasons discussed above) that 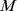 is large and discretize the recruitment process into 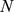 landmark events. As a consequence we can only represent shortcuts between these landmark events and not between the intermediate states in our computer model. The probability for a shortcut 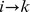 to be traversed is then given by
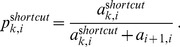
(12)


We ask how a small number of long-range transitions affect the ChIP signal and how such shortcuts can be detected. Our computer experiments show that processes with shortcuts show distinct dynamics from pure SR processes. However, usually the recruitment process is not known a priori, i.e. all that is given are ChIP time-series 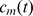, and it is difficult to determine the underlying recruitment scenario from this data alone. How and to what extent it is possible to distinguish the two scenarios from data is discussed in the next section.

## Results

Using various recruitment scenarios, we now show how shortcuts can be detected in a recruitment process using the GRM. The initial distribution 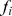 is crucial for the detection of these transitions. A synchronized, peaked initial distribution for a cell population obtained at the time of transcription initiation does not directly allow to infer the existence of shortcuts. The 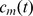
’s show oscillations in both cases. Given the oscillating 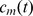 alone therefore does not allow to infer whether the source of the signal is SR or PR. However, a de-synchronized initial condition represents the equilibrium distribution of the SR process but not the equilibrium distribution of a PR process. With this type of initial condition oscillations can only be observed for PR.

**Figure 3:**
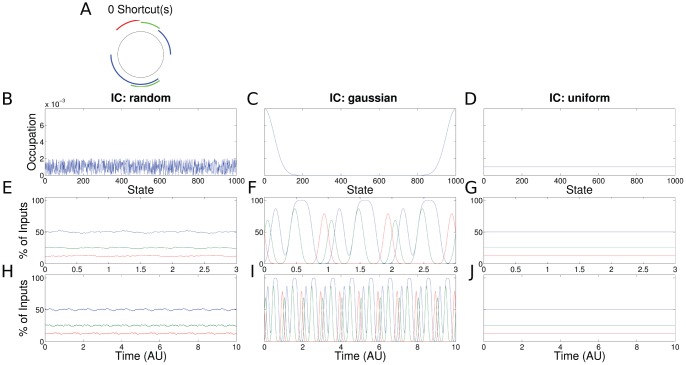
Results for the SR model for the three initial conditions and no shortcuts. (a) Recruitment network of circularly linked states (black), the binding of the proteins associated with the states by the matrix 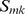 are visualized in blue, green and red. The columns indicate different initial conditions. 1^st^ column: randomly distributed initial condition, 2^nd^ column: Gaussian distributed initial condition, 3^rd^ column: uniform initial condition. (b-d) The three types of initial conditions. (e-g) Predicted ChIP signal for three proteins (arbitrary time unit). (h-j) The same ChIP signal for a longer time interval. While the Gaussian initial condition (synchronized cell population) results in periodic dynamics for the ChIP signal (e,f), initial conditions that represent the de-synchronized cell populations show a constant ChIP signal.

To demonstrate this fact, we consider three types of initial conditions in our in silico experiments: (i) A de-synchronized cell population with a randomly distributed initial condition (Fig. 3b), (ii) an (imperfectly) synchronized cell population represented by a Gaussian initial condition (Fig. 3c), and (iii) a de-synchronized cell population with a uniform initial condition (Fig. 3d).

In simulations we consider three different proteins in a hypothetical recruitment process. By linking these proteins to specific states using the 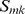 matrix (e.g. Fig. 1a), we can calculate the resulting concentration 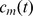 for these proteins that are measured in the in silico ChIP experiment.

Lemaire et al. (2006) argue that more than 100 histone and protein modifications occur during the recruitment process and transcription complexes can contain more than 50 proteins and estimated that about 200 distinct protein complexes that form on the promoter. With this in mind, we use 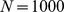 for the discretization in our simulations for the recruitment process, while 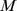 is considered to be large. The transition rates from a state 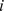 to its successor state 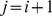 are chosen to be 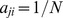 (Fig. 1d). A different value would trivially rescale the period 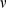 as discussed above.

Figure 3 shows the three initial conditions (Fig. 3b,c,d) and the time series 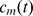 of the hypothetical proteins (Fig. 3e,f,g: short-term dynamics, Fig. 3h,i,j: long-term dynamics). The circular recruitment network is shown in Fig. 3a in black, where the blue, green and red bars indicate the protein binding patterns defined by the matrix 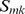. The ChIP signal exhibits cyclical behavior for the Gaussian initial condition. No decay of amplitudes is detectable. In case of the randomly distributed initial condition the ChIP signal shows recurrent noisy characteristics. For the uniform initial condition the predicted ChIP signal remains constant, as expected.

**Figure 4:**
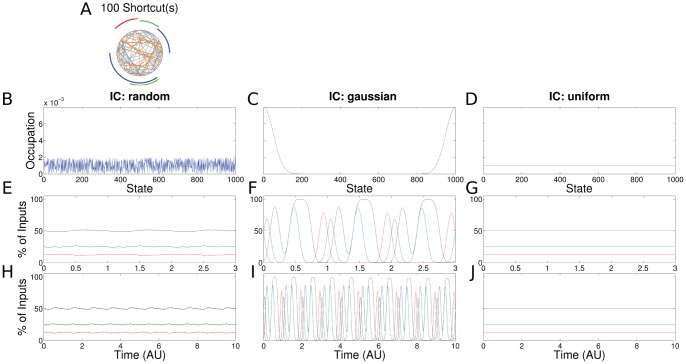
Results for the PR with 100 transitions with low transition rates (same setup as in Fig. 3). (a) The transitions branching backward and forward are shown in blue and orange, respectively. A distinction with the ChIP signal of a SR process (Fig. 3) is practically not possible.

**Figure 5:**
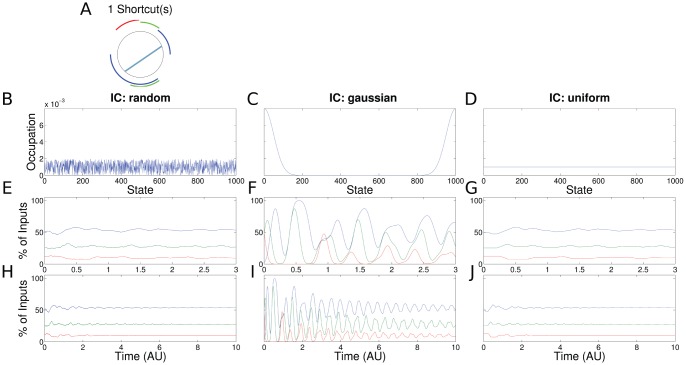
Results for the PR with one shortcut (same setup as in Fig. 3). (a) The shortcut (branching backward, 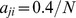) is shown in blue. (f) In case of a synchronized cell population (Gaussian initial condition) the ChIP signal exhibits periodical dynamics similar to the case of SR (Fig. 3f). (h,j) The initial conditions that represent de-synchronized cell populations show initial oscillating dynamics.

Next, we consider a PR process that is obtained from a SR process by adding many random transitions with low transition rates (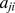 uniformly distributed in 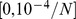, Fig. 4). The ChIP signal for all three initial conditions is indistinguishable from the ChIP signal of the SR process. This changes if the recruitment process includes shortcuts, that is long-range transitions. In Fig. 5 we show an example with one shortcut that branches backward (with a transition probability of 
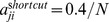
). For the Gaussian initial condition, the ChIP signal exhibits cyclical behavior with decaying amplitude. With this initial condition the process would therefore not be distinguishable from a SR process with non-negligible diffusion constant, i.e. where 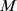 is not large. As we see in the 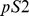 example below, it is also possible to have processes with shortcuts that do not show notable decay. As a consequence notable decay of the ChIP signals can already hint at shortcuts but it does not prove their existence. In contrast to the recruitment scenario without shortcuts (Figs. 3 and 4), the two de-synchronized initial conditions (random and uniform) also show an oscillating (and decaying) ChIP signal. This allows to distinguish the SR processes with diffusion from the PR processes with shortcuts by using a de-synchronized initial condition.

**Figure 6:**
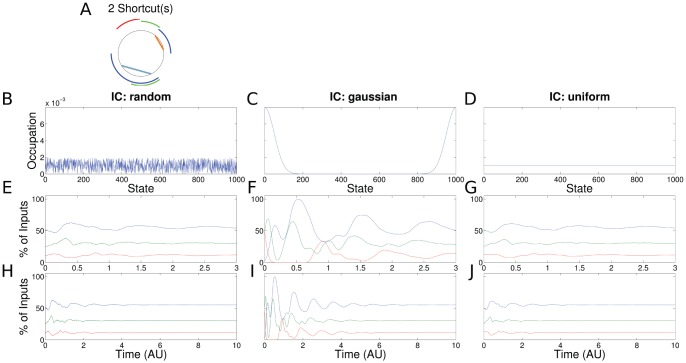
Results for the PR with two shortcuts (same setup as in Fig. 3). (a) The shortcuts branching backward and forward are shown in blue and orange, respectively. (f) The periodicity of the recruitment process is reflected in the ChIP signal for a synchronized cell population. (e,g) The de-synchronized cell populations show initial oscillatory dynamics with decaying amplitude.

The results for a PR process with two shortcuts (one of the transitions branches forward and the other backward) and the scenario with one shortcut behave similarly (Fig. 6, 
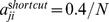
). Uniform initial conditions show an oscillating signal in the beginning and decay quickly to the steady state. Note that the final steady state of the ChIP signal does not depend on the initial condition. Only in the transient phase informative dynamics can be observed.

**Figure 7:**
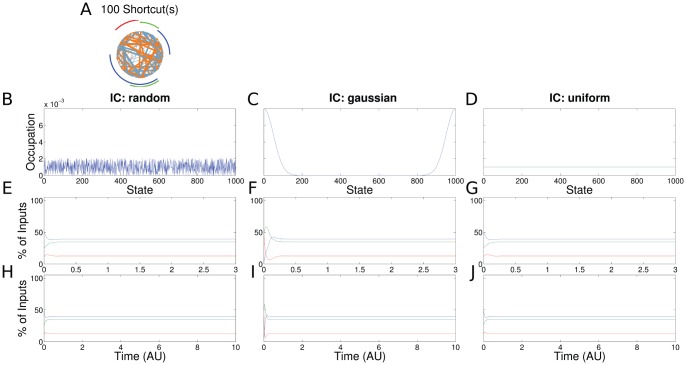
Results for the PR with 100 shortcuts (same setup as in Fig. 3). (a) The shortcuts branching backward and forward is shown in blue and orange, respectively. The thickness of the lines corresponds to the transition rate of the shortcuts (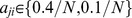). (e-g) The ChIP signal converges rapidly to a constant value for all initial conditions.

Next, we consider a PR process that contains 100 randomly drawn shortcuts (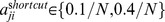). As seen in Fig. 7 the ChIP signals reach a stationary state almost immediately for all initial conditions. This indicates that highly random recruitment processes like this do not show cyclical ChIP signals in any initial condition.

**Figure 8:**
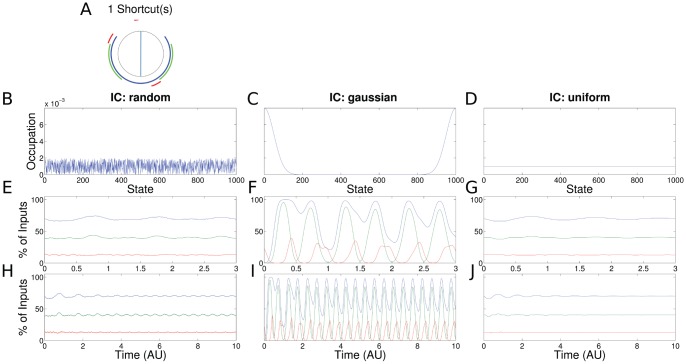
Results for the PR for transcription of the 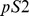 gene (same setup as in Fig. 3). (a,e–j) 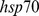 (red), 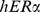 (green), 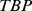 (blue). The predicted ChIP signal (f) and the experimental ChIP (see Fig. 9a for details) have the same characteristics.

**Figure 9:**
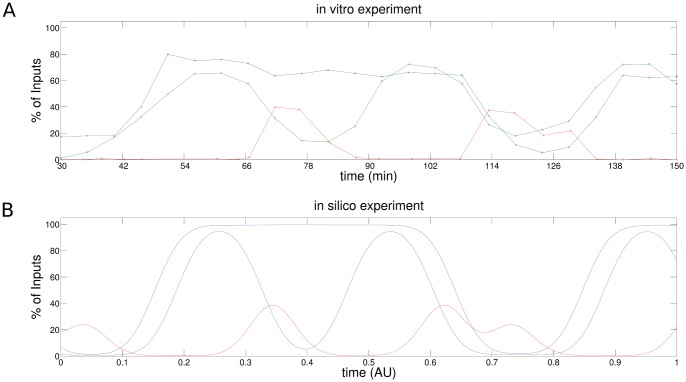
Result of ChIP experiments for 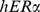-induced transcription of the 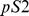 gene. (a) ChIP signal of in vitro experiment (data extracted from Metivier et al. (2003)) (b) ChIP signal in silico experiment using the GRM (see Fig. 8). The ChIP signals of both experiments exhibit the same characteristics.

Finally, we present an example which shows that interpretation of ChIP data can be ambiguous, by assuming a certain characteristic of the underlying recruitment process. The example demonstrates that for this experimental set-up it is not possible to decide whether a SR or a PR process is present using only the synchronized initial condition. The analysis of the ChIP data by Metivier et al. (2003) for the transcription of the 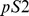 gene was based on the assumption that the recruitment process is strictly sequential. According to their interpretation of the data, after an unproductive initiation cycle there is an alternation between even and odd transcription cycles: In odd cycles the promoter is cleared completely, whereas in even cycles two general transcription factors (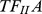 and 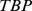) remain bound to the promoter. To test whether this strict distinction between even and odd cycles is the only possible interpretation of the data, we simulate recruitment with a PR process using the GRM: A shortcut enables the cell to clear the promoter completely after an odd cycle by chance (
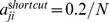
, Fig. 8a) or the cell can proceed with an even transcription cycle with bound 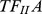 and 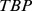. The aim is to analyze if the alternation of the transcription cycles needs to be deterministic or can be probabilistic. As illustrated in Fig. 9 the experimental ChIP data by Metivier et al. (2003) and the resulting synthetic ChIP data based on probabilistic promoter clearance show the same characteristics: The periodicity of the TBP concentration is twice as long as the periodicity for 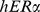. Another feature that both ChIP signals exhibit is the alternating periodicity in the 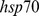 ChIP signal. Thus a distinction whether this process is SR or PR is not possible from the ChIP data with synchronized initial condition. However, with a de-synchronized initial condition this distinction is possible (compare Fig. 8).

## Discussion

The interpretation of ChIP time-series data and the resulting order of protein binding in complex formation relies on the correct assumption of the characteristics (SR/PR) of the underlying recruitment process. An incorrect assumption about this characteristics will result in false or distorted protein binding patterns.

We showed that in a recruitment process many random transitions with low transition rates do not notably change the ChIP signal. Short-range transitions in circular recruitment processes – in particular reversible transitions – merley change the frequency of such processes (if 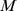 is large). This shows that hypotheses explaining random binding events of proteins observed in GFP experiments as “non-functional” [17], [24] are compatible with short-range modifications of SR processes which do not interfere with the sequential characteristic of the recruitment process on average.

If random transitions are sufficiently long-range, this implies a non-negligible value for the diffusion constant of the process. Yet, non-negligible diffusion could still mean that the number of intermediate states 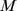 in a sequential scenario is not sufficiently large. Signals showing diffusive decay may therefore either indicate long-range transitions or a low number of intermediate states between landmark states.

Our examples show that by using synchronized initial conditions SR processes and PR shortcut processes are hardly distinguishable. To distinguish these two possibilities from each other one may note that for PR processes a de-synchronized initial condition does not represent the stationary state. However, this initial condition does represent the stationary state for SR processes. Thus, for a de-synchronized initial condition, oscillations in the ChIP signal indicate the presence of a PR process with shortcuts. Whether de-synchronized initial conditions produce oscillations or not is an indicator to distinguish between these two types of recruitment processes as long as the number of shortcuts is not too large. If the number of shortcuts is large, any initial condition will decay to the steady state extremely fast.

Note, that this method requires a sufficient temporal resolution for the ChIP measurement to ensure the detection of oscillations in the initial transient phase. The accuracy and signal-to-noise ratio of the ChIP measurement also affects the reliability of this method. If the transition rate of shortcuts in a PR process is too low or the transition is too close to short-range the presented method to distinguish SR from PR processes will yield no conclusive result. Yet, under those conditions the process can *effectively* be regarded as “SR”.

The de-synchronized initial condition can be implemented experimentally for example by splitting a cell population into many sub-populations. The transcription of each sub-population is then triggered randomly (i.e. varying amounts of estradiol are applied to the sub-populations at random times in case of the 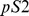 gene). When mixing the sub-populations again, the resulting cell population has approximately the desired de-synchronized distribution.

In previous work [10], [27] we showed that sequentially linear models are able to reproduce the characteristics of SR processes. The GRM presented in this paper enables us to formulate an interpretation of the interaction rates in these gene regulatory network models: These interaction rates do not describe the characteristics of the direct interaction of proteins. Instead these interaction rates must be perceived as “affinity rates” in a broader sense. This affinity does not necessarily mean a direct physical interaction of proteins. Instead, they describe how a protein affects the affinity of another protein to the protein complex on the promoter site.

### Conclusions

In this paper we introduced a generalized recruitment model that is capable of representing various recruitment scenarios ranging between strictly sequential recruitment (SR) and completely probabilistic recruitment (PR). In our analysis we have shown that for a wide range of probabilistic transitions (short-range transitions and transitions with sufficiently low rates) the results of GFP and ChIP experiments can be reconciled. These transitions do not fundamentally change the sequential characteristic of the recruitment process. Where PR and SR scenarios cannot be reconciled (shortcuts), they also are distinguishable.

We have proposed an experimental test that makes it possible to check the characteristic of the recruitment process. A low number of shortcuts can be detected by observing oscillatory signals when using de-synchronized initial conditions. If the probabilistic characteristics of the recruitment processes dominates (many shortcuts), then the ChIP signals converge quickly to a steady state for all initial conditions.

We have illustrated this problem for the 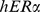-induced transcription of the 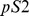 gene. We have shown for this recruitment process that the available data can be explained by either alternating or probabilistic promoter clearance. The proposed experimental test using de-synchronized initial conditions could be used to decide between both possibilities.
